# Substantial tumor regression in prostate cancer patient with extensive skeletal metastases upon Immunotherapy (APCEDEN): A case report

**DOI:** 10.1097/MD.0000000000018889

**Published:** 2020-02-21

**Authors:** Bandana Sharan, Srikanth Chiliveru, Jasmine Bagga, Sakshi Kohli, Asmi Bharadwaj, Ashok K. Vaid, Chaitanya Kumar

**Affiliations:** a APAC Biotech Pvt Ltd, Gurgaon, Haryana, India; b Department of Medical Oncology and Hematology, Medanta - The Medicity, Gurgaon, Haryana, India.

**Keywords:** APCEDEN, dendritic cells, immunotherapy, interferon gamma, neutrophil lymphocyte ratio, platelet lymphocyte ratio, prostate carcinoma, regulatory T cells

## Abstract

**Rationale:**

Prostate cancer along with colorectal and lung cancers accounts for 42% of cancer cases in men globally. It is the first cancer indication for which the use of active immunotherapy, Sipuleucel-T (Provenge) was granted by the FDA in 2010. This study presents a case of prostate carcinoma and the tumour remission observed after administration of a personalised Dendritic cell vaccine (APCEDEN).

**Patient concerns:**

A 58 years old Caucasian male diagnosed with prostate carcinoma with GLEASON score 8. The patient had previously been diagnosed with Renal Cell Carcinoma (RCC) in 1996 and had undergone nephrectomy of the right kidney. PET CT scan revealed multiple intensely PSMA avid lesions noted in both lobes of the prostate gland with SUVmax −28.3 and the prostate gland measuring 3.2 × 3.2 cm displaying maximum dimensions.

**Diagnosis:**

FNAC followed by PETCT confirmed CA Prostate and further supported by increased serum PSA level.

**Interventions:**

The patient underwent personalised Dendritic Cell Immunotherapy APCEDEN regimen of six doses biweekly, in a time frame of 3 months were given both via intravenous and intradermal route. Six months post completion of APCEDEN, the patient was administered 6 booster shots for 6 months.

**Outcomes:**

Progressive remission of carcinoma was observed along with reduction in PSA and Testosterone levels. PET CT showed decline in PSMA avidity by 50% with SUVmax −14.0 and normal size and shape of prostate gland.

**Lessons:**

Prostate carcinoma is the second most common cancer in men with majority of them exhibiting locally advanced disease. Apparently 20% to 30% of them are categorized as relapsed cases after various therapeutic interventions. Modulating immune system is an emerging therapy termed as Immunotherapy and potentiates the killing cancer cells via immune activation. Interestingly, prostate cancer is slow growing and it provides the scope and time to mount an anti-tumor response which makes it an attractive target for immunotherapy. This case study demonstrates the efficacy of APCEDEN Immunotherapy regimen resulting in a significant disease remission benefiting the patient.

## 1 Introduction

The two arms of immune system: innate and adaptive immunity work in a regulated manner to maintain a state of equilibrium or homeostasis, essential to control tumor development. Dysregulated immune system sets a favorable niche for the unchecked cell proliferation in the body.^[1]^ Cancer is characterized by the accumulation of a variable number of genetic alterations resulting in mutations and the loss of normal cellular regulatory processes.^[2]^ These altered genetic events result in the expression of neoantigens, differentiation antigens, or cancer testis antigens, which can lead to presentation of peptides bound to major histocompatibility class I (MHCI) molecules on the surface of cancer cells, distinguishing them from their normal counterparts. These neoantigens are sensed by specialised Antigen presenting cells (APCs), which in turn process and present the antigen to the effector T lymphocytes and ultimately directs whole immune system to work in tandem for the removal of the tumor cells expressing the mutated antigen.^[3]^ Multiple factors contribute in eliciting the immune response including stimulatory and inhibitory signals that lead to immune cell activation or suppression.^[4–8]^

Cancer epidemiology suggests prostate cancer to be the second most common cancer in men with an estimated incidence of 1.1 million patients annually.^[9]^ The majority of these patients exhibit locally advanced disease. Furthermore, 20% to 30% of them are categorised as relapsed cases after therapies with curative intent.^[10,11]^ Metastatic Prostate cancer (Pca) has been classified into hormone sensitive (HRPC) and castrate refractory (CRPC).^[12]^ The median overall survival for CRPC ranges from 12.2 to 34.7 months as indicated by a recent phase 3 study.^[13–17]^ Various treatment options are available for Pca such as hormonal therapy, chemotherapy, and radiotherapy showing significant improvements in overall survival. Redirecting or modulating the immune system is emerging as an interesting as well as very alluring option for the development of anticancer treatment. Immunotherapy potentiates the killing of malignant cells by the components of immune system. There are different available types of immunotherapy, which includes immune checkpoint inhibitors, antibodies, vaccines, adoptive cell transfer. Interestingly, prostate cancer is slow growing and it provides the scope and time to mount an anti-tumor response^[18,19]^ which makes it an attractive target for immunotherapy. With the approval of SIPULEUCEL-T by US FDA, cell based vaccines also known as active immunotherapy that augments the capacity of immune cells to target tumor cells, are emerging strongly as a promising immunotherapeutic branch. SIPULEUCEL-T is an autologous and personalized dendritic cell based immunotherapy product and got approval for treatment for prostate cancer after successful completion of phase 3 trial in CRPC patients.

Various other autologous dendritic cell based products with different immune modulations are under investigation and clinical trials for validating their efficacy. Recently, an Indian biotechnology company has received commercial approval for their autologous dendritic cell based product APCEDEN on the basis of their phase 2 trial on multiple chemo failure, refractory and multiple metastatic cancer patients.^[20,21]^ Here, we describe a case of prostate cancer and the significant improvement after receiving personalised Dendritic cell based immunotherapy APCEDEN.

## 2 Methods

### 2.1 APCEDEN preparation

The growth factor (G-CSF) is infused into the patients before leukapheresis (Cobe Spectra, Terumo BCT) to mobilize the new cells from bone marrow. A total of 100 mL volume of Peripheral blood mononuclear cells (PBMCs) were collected and monocytes were separated using plastic adherence method. Monocytes were differentiated using GM-CSF (50 ng/mL, R&D System, GMP grade) and IL-4 (10 ng/mL, R&D System, GMP grade) and AIM V medium with added 2 mM glutamine (GMP Grade, Thermo fisher Bioscience). The culture was replenished with complete medium on 3rd and 5th day. On the sixth day, tumor lysate (2 mg/mL) was exposed to immature dendritic cells and 5 mg/mL Poly I: C (Invitrogen) was used as maturation stimuli. On the eighth day of culturing, mature dendritic cells were harvested and subjected to quality control which included endotoxin assay, mycoplasma assay, sterility assay, immuno-phenotyping of mDC, viability assay, and mixed lymphocyte assay. The mDCs tested negative for Endotoxin (QCL1000, Lonza), Mycoplasma (Mycolert, Lonza), were sterile (post 14 days of culturing) with a viability of >95% (7AAD PerCP). The distribution of markers was >89.62% (CD83, FITC), >96.39% (CD80, PE), >87.50% (CD86, SB600), >49.9% (CCR7, FITC), >83.0% (HLA DR, PacOrgange), >76.73% (CD205, FITC), >85.24% (CD209, PE), >81.4% (CD40, SB436) as seen by Flow Cytometry with MLR assessment >2.5 folds.

### 2.2 Antigen preparation

Ultra sound guided 21 core CA prostate biopsy collected in Tissue transported medium (Nutriprep) from the subject was received and was further histo-pathologically confirmed for its malignancy. It weighed (480 mg) and was processed further for homogenization. Homogenized tissue was subjected to six repeated freeze (−196) thaw (37) cycles, and further the protein was quantified by Bradford method.

### 2.3 NLR and PLR

The hemogram assessment of the patient 15 months post APCEDEN therapy was done to determine neutrophil lymphocyte ratio (NLR) and platelet lymphocyte ratio (PLR). The NLR was defined as a simple ratio between the absolute neutrophil count and the absolute lymphocyte count; similarly, PLR was defined as the ratio of the absolute platelet count and the absolute lymphocyte count.

### 2.4 Treg and IFNγ

The peripheral blood samples at baseline and vital stages of the therapy until 17 months post APCEDEN therapy were analysed for biogenesis of T regulatory (Treg) cells and intracellular Interferon gamma (IFNγ). The flow cytometric gating strategy followed was CD3/CD4 and CD8/CD25/CD127 for T regulatory cells and CD3/CD4 and IFNg for Interferon gamma, respectively.

### 2.5 Ethical review

The authors have obtained appropriate institutional ethical review board approval in this case from the ethics committee of Medanta—The Medicity Hospital, Gurgaon, India and have followed the principles outlined in the Declaration of Helsinki for all human experimental investigations. In addition, for all the investigations done in this study, written informed consent has been obtained from the patient.

### 2.6 Case presentation

**Figure 1:**
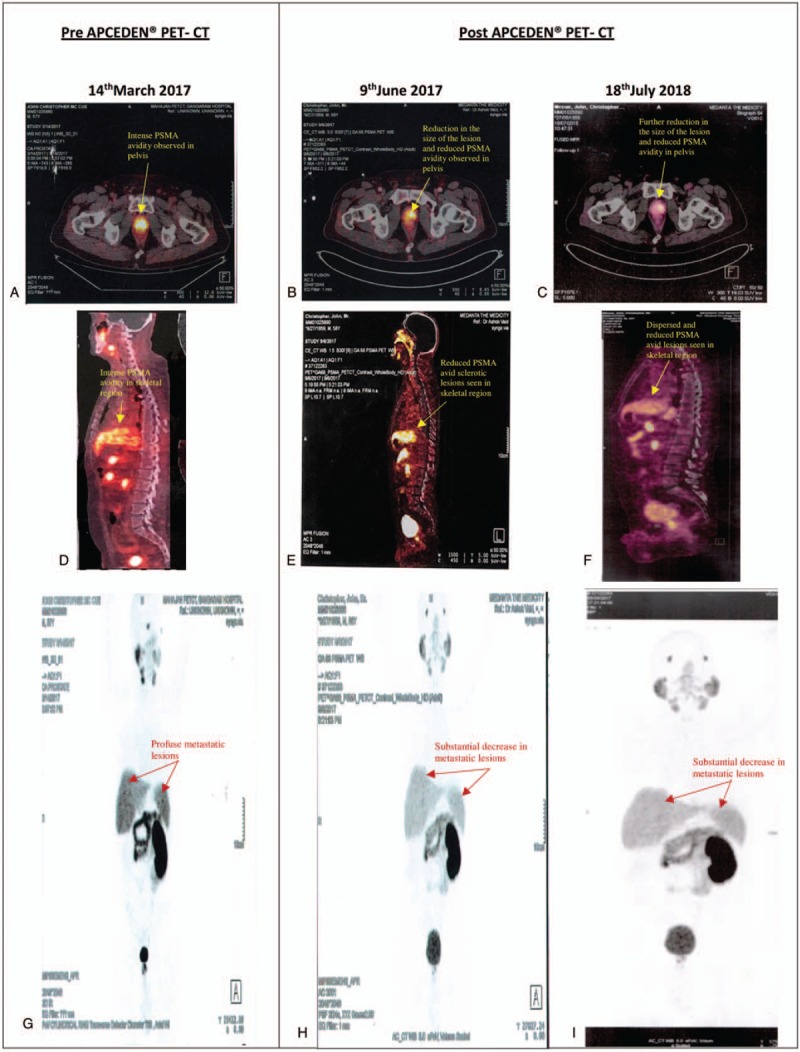
Comparison of PET-CT scan of the patient on March 14, 2017 before APCEDEN therapy with PET-CT scans done on June 9, 2017 post APCEDEN therapy and July 18, 2018 approx. 13 months after last dose of APCEDEN. (A)–(C) are PET scan images of the pelvis region displaying reduced PSMA uptake and shrinkage in lesions post APCEDEN treatment. (D)–(F) are PET scan images of the skeletal region showing reduced and dispersed PSMA avid sclerotic lesions at the both the time points respectively post APCEDEN treatment. (G)–(I) are full body PET scan images demonstrating a substantial tumor regression of metastatic lesions.

This report presents a case of 58 years old Caucasian male, a case of confirmed prostate carcinoma with GLEASON score 8 diagnosed in August 2014 ruled out through FNAC followed by PET CT. The subject received chemotherapy (Zytiga and Lupron) and hormonal treatment, but represented a refractory pattern after multiple chemo failures, with PSA levels rising above 200 ng/mL. The patient had previously been diagnosed with Renal Cell Carcinoma (RCC) in 1996 and had undergone nephrectomy of the right kidney. With a history of recurrences, he decided to opt for active immunotherapy. PET CT scan prior to the immunotherapy revealed multiple intensely PSMA avid lesions noted in both lobes of the prostate gland with SUVmax −28.3 and the prostate gland measuring 3.2 × 3.2 cm displaying maximum dimensions representing primary mitotic lesions and furthermore showing few mildly avid sub-centrimetric para aortic, aortocaval, and bilateral external iliac lymph nodes. Post-administration of Dendritic Cell Immunotherapy APCEDEN (6 doses between March 2017 and June 2017), remission of the carcinoma was observed as indicated by the significant decline in the PSMA avidity by 50% reduction in SUV max dwindling to 14.0 regaining the normal size and shape of the prostate gland with no obvious focal lesions, consistent with primary disease and likewise no significant PSMA uptake in the iliac and pelvic lymph nodes (Fig. 1). Six months post completion of APCEDEN, the patient was administered 6 booster shots of APCEDEN, once monthly from January 2018 until June 2018. PET CT scan in July 2018 revealed the Prostate to be normal in size and no other PSMA expressing lesions were noted elsewhere to suggest a metastatic disease.

**Figure 2:**
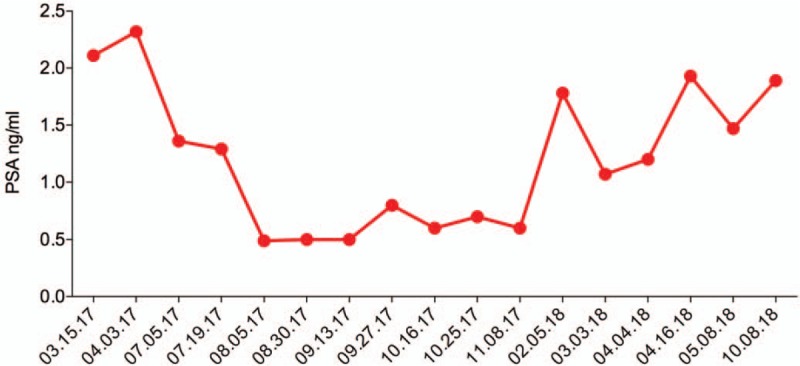
PSA levels (ng/mL) over a period of nine months post receiving last dose of APCEDEN.

The progressive remission of the carcinoma was seen in concordance with the blood PSA and Testosterone levels. The biochemical analysis was done over a period of nine months starting July 2017, indicated a stability and reduction in PSA levels with the lowest of 0.4 ng/mL in August 2017 (2 months post APCEDEN) (Fig. 2). Similarly, the testosterone levels displayed a comparable pattern of reduction with the lowest of 8.9 ng/dL at the same time point in August 2017.

**Figure 3:**
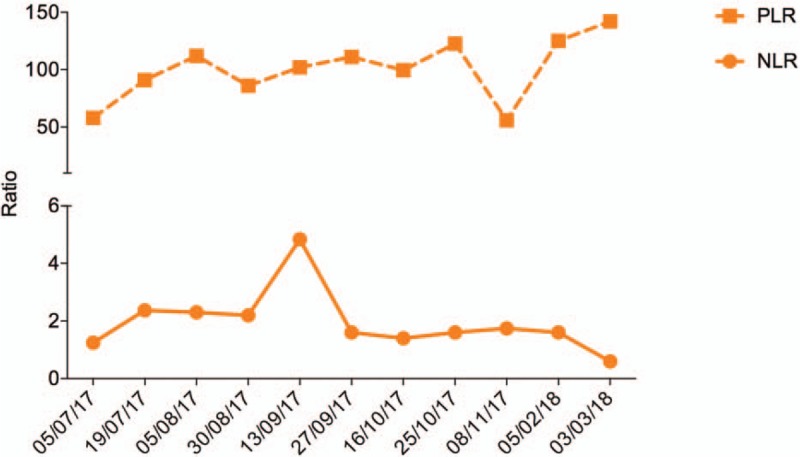
Neutrophil lymphocyte ratio (NLR) and platelet lymphocyte ratio (PLR) as a prognostic marker for immunotherapy. The orange line indicates NLR and dotted orange line indicates PLR.

The prognosis of immunotherapy was estimated by NLR and PLR through peripheral blood hemogram assessment at regular intervals over a period of 15 months starting from July 2017 (1 month post-therapy) (Fig. 3). NLR and PLR are considered as independent robust response biomarkers of therapeutic efficacy of a vaccine against cancer. The NLR values showed an early sharp reduction of as low as 1.25 in July 2017 (1 month post-therapy), indicating an immediate post-therapeutic effect followed by a median lowest value of 1.4 in October 2017 (4 months of post-therapy). The NLR value was maintained at <1.5 until July 2018 (13 months post-therapy). The PLR values also followed a similar pattern with a value of 58 immediately post-therapy, a median value of 86 in August 2017 (2 months post-therapy) and the lowest value of 56 in November 2017 (5 months post-therapy). The value was maintained <100 until 13 months post-therapy (July 2018). The observed decline in the values of both the biomarkers suggested a positive prognostic value of APCEDEN.

**Figure 4:**
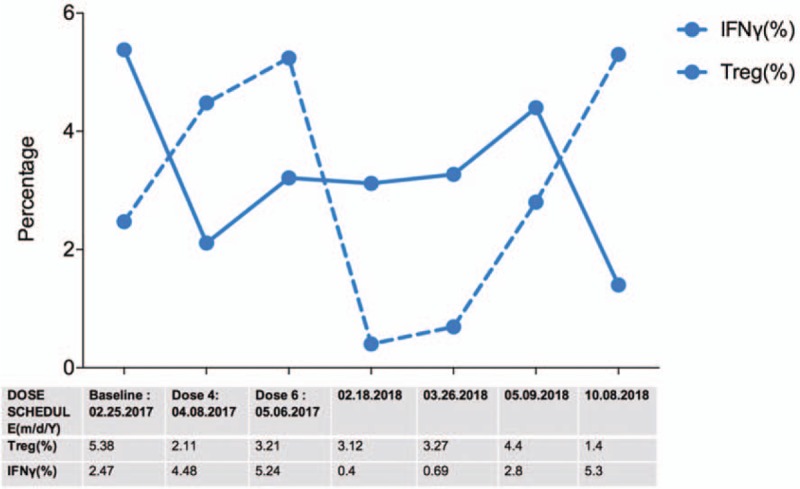
Immune assessment using percentage of T regulatory and IFNγ producing T cells as a therapeutic efficacy marker for APCEDEN. The blue line indicates decreasing Treg levels and dotted blue line indicates increasing levels of IFNγ producing cells. All data points are percentages of the immune cells.

The efficacy of APCEDEN and the immune response of the patient were further investigated by the immune cell assessment using peripheral blood at different time points of the treatment regimen. Biogenesis of T regulatory (Treg) cells and intracellular Interferon gamma (IFNγ) expression in T lymphocytes are a paradigm of WHO criterion for therapeutic efficacy of cancer drugs in accordance to the Response Evaluation Criteria in Solid Tumors (RECIST) and Immune-Related Response Criteria (irRC). As compared to the baseline value (pre APCEDEN therapy) a substantial decrease in Treg cell percentage was observed after dose four of APCEDEN, which gradually declined till sixth dose of APCEDEN. On the contrary, a reverse correlation was observed with IFNγ levels that were induced post APCEDEN with the maximum of 5.24% at dose six of the therapy. Furthermore, the 6 booster shots contributed to the sustained immunological response with APCEDEN maintaining the antagonistic levels of the two immune markers till August 2018, the last time point examined (Fig. 4). Patient has provided informed consent for publication of the case.

## 3 Discussion

In this paper, we report a case study of a patient suffering from metastatic refractory prostate carcinoma. After exhausting conventional treatments including multiple chemotherapies and hormone therapy, the patient was administered autologous Dendritic Cell immunotherapy APCEDEN. Although other immunotherapies such as immune-check point inhibitors were also an available option, the poor performance status of the patient coupled with compromised mobility did not qualify for these drugs to be chosen. Moreover, with negligible side effects associated with APCEDEN due to its autologous nature and its due approval by the Indian FDA for use in refractory cases of multiple indications with CA prostrate being one of them, APCEDEN treatment was initiated. The present study indicated tumor lysate loaded dendritic cell vaccine to be completely safe and suggestively effective to treat metastatic prostate cancer, as revealed by the post therapy follow up PET CT scan. We report an overall survival benefit of 20 months with APCEDEN with the patient still alive (February 2019). Furthermore, in concordance with the survival benefit and improved quality of life score for patients in the conducted phase 2 trial of APCEDEN, the patient in the present study experienced improvement in quality of life and an increased appetite.^[21]^ The subject in this case report responded positively to immunotherapy second time in a row; as he suffered from kidney cancer 21 years ago and got cured with TVI-Kidney-1 (Tvax Biomedical, Olathe, KS). Following diagnosis of prostate cancer in the year 2014, his disease metastasized in his bone and multiple nodes which led him to opt for administering APCEDEN in the year 2017. He was facing shortness of breath as well as compromised quality of life. He responded to immunotherapy well as revealed from the PET CT scan done after he completed his six doses of APCEDEN regimen.

Immunotherapy is designed to boost the body's natural defenses to fight the cancer. It uses materials made either by the body or in a laboratory to improve, target, or restore immune system function. There are set of patients who respond to immunotherapy very well and recent researches have connected gut microbiota composition with treatment efficacy of immune checkpoint inhibitors in melanoma patients.^[22,23]^ Additionally, a meta-analysis by conforti et al, established difference response of men and women towards immunotherapy with women showing a better overall survival than men.^[24]^ Thus, it becomes very vital to understand the underlying tumor–host immune interactions resulting in better response to immunotherapy to provide maximum benefits of the treatment. In this case study, the subject's response to immunotherapy is remarkable but no understanding for correlating it with genetic/metabolic factors has been provided, as this involved a comprehensive genetic comparison of the responder versus non-responder and it is out of scope of this case study. The study once again highlights the safety and efficacy of APCEDEN, a completely customised cancer vaccine with great potential for treatment of advanced prostrate carcinoma. A lot of recent studies are now focussing on a “combinatorial approach” for treating prostrate carcinoma using both check-point inhibitors as well as dendritic cell based immunotherapies to enhance the overall survival of advanced prostate cancer patients.

## Acknowledgments

Not applicable.

## Author contributions

**Conceptualization:** Bandana Sharan, Chaitanya Kumar.

**Data curation:** Bandana Sharan, Srikanth Chiliveru, Jasmine Bagga, Sakshi Kohli, Asmi Bharadwaj, Ashok K Vaid, Chaitanya Kumar.

**Formal analysis:** Bandana Sharan, Srikanth Chiliveru, Jasmine Bagga, Sakshi Kohli, Asmi Bharadwaj, Ashok K Vaid, Chaitanya Kumar.

**Investigation:** Bandana Sharan, Srikanth Chiliveru, Jasmine Bagga, Sakshi Kohli, Ashok K Vaid, Chaitanya Kumar.

**Methodology:** Bandana Sharan, Srikanth Chiliveru, Jasmine Bagga, Sakshi Kohli, Chaitanya Kumar.

**Project administration:** Asmi Bharadwaj, Ashok K Vaid, Chaitanya Kumar.

**Resources:** Asmi Bharadwaj.

**Supervision:** Bandana Sharan, Srikanth Chiliveru, Jasmine Bagga, Sakshi Kohli, Asmi Bharadwaj, Ashok K Vaid, Chaitanya Kumar.

**Validation:** Bandana Sharan, Srikanth Chiliveru, Jasmine Bagga, Sakshi Kohli, Asmi Bharadwaj, Ashok K Vaid, Chaitanya Kumar.

**Visualization:** Bandana Sharan, Srikanth Chiliveru, Jasmine Bagga, Sakshi Kohli, Ashok K Vaid, Chaitanya Kumar.

**Writing – original draft:** Bandana Sharan, Chaitanya Kumar.

**Writing – review & editing:** Bandana Sharan, Srikanth Chiliveru, Chaitanya Kumar.
